# Influence of sugar beet (*Beta vulgaris*) pulp on *in vitro* fermentation kinetics, methanogenesis, and total gas production of wheat straw in buffalo (*Bubalus bubalis*)

**DOI:** 10.3389/fvets.2026.1927413

**Published:** 2026-08-20

**Authors:** Avijit Dey, Shubham Thakur, Sanjay Kumar, Tirtha Kumar Datta

**Affiliations:** 1 Division of Animal Nutrition and Feed Technology, ICAR-Central Institute for Research on Buffaloes, Hisar, India; 2 Division of Animal Genetics and Breeding, ICAR-Central Institute for Research on Buffaloes, Hisar, India

**Keywords:** enzyme activities, feed digestibility, methane emissions, roughage diet, rumen fermentation, sugar beet pulp, volatile fatty acids

## Abstract

**Introduction:**

Wheat straw (WS) is one of the major crop residues and valuable feed resources for livestock reared by the resource-poor farmers of many Asian countries. Sugar beet (*Beta vulgaris* L.) is a cash crop produced all over the world for sugar production by the industries. Strategic supplementation of sugar beet pulp (SBP) to a WS-based diet could modulate rumen fermentation by supplying highly fermentable carbohydrates to rumen microbes, thereby improving utilization of WS. Therefore, the current study aimed to examine the influence of SBP in modulating rumen fermentation kinetics, volatile fatty acid production, enzyme activities, and methanogenesis of WS using rumen liquor of water buffalo.

**Methods:**

*In vitro* fermentation kinetics was conducted using the automatic gas production system for 72 h at 39 °C with rumen liquor of buffalo.

**Results:**

Supplementing SBP either at 10% or 20% to WS enhanced (*p* < 0.05) total gas production, potential gas production (*b*) and rate (per h) of gas production (*c*). Despite the enhanced (*p* < 0.05) true degradability of dry matter, organic matter digestibility, and metabolizable energy content of the composite feeds supplemented with SBP, methanogenesis was comparable (*p* > 0.05) to that in the control. The supplementation of SBP also enhanced (*p* < 0.05) volatile fatty acid production and fibrolytic enzyme activities (CMCase and xylanase).

**Discussion:**

This study demonstrates that SBP, a widely available by-product of the sugar industry, could be an advisable choice to enhance the utilization of WS, thereby improving livestock productivity.

## 1 Introduction

Crop residues, such as wheat/rice straws, sorghum/maize stovers, and sugarcane tops/bagasse, are suitable feed resources for ruminants reared by resource-poor farmers of many Asian countries. They are very rich in cellulose and hemicellulose; however, their high lignin content makes structural carbohydrates less available to ruminal microorganisms, resulting in poor nutritive value of the feeds (1, 2). Many strategies have been undertaken to improve the feeding value of these crop residues for sustainable animal production. As these crop residues have meagre fermentable nutrients, supplementation with protein- or carbohydrate-rich feeds is an option to enhance their utilization as animal feed and reduce environmental pollution by minimizing wastage (3–5).

Asia is blessed with the largest ruminant population in the world, with India as the leader, having 193.46 million cattle and 109.85 million buffalo, besides 74.26 million sheep and 148.88 million goats (6). However, the productivity of animals in the region is very low owing to poor genetic heritage and limited and low-quality feed resources (7). Buffalo (*Bubalus bubalis*) plays a significant role in improving socioeconomic development of Asian countries. Regarded as ‘*Black Gold*’, buffalo is the backbone of milk and meat production in the region (8). Buffaloes have several microbial populations that help sustain their production in different environments. Buffaloes are also less susceptible to many diseases (9) and better converters of poor-quality feed resources than cattle (10). WS serves as the main roughage source for sustaining animal rearing by the farmers of Asian countries, including India. Interestingly, in Italian specialized systems, the coexistence of dairy buffaloes and durum wheat (*Triticum durum* Desf.) on the same farms ensures straw availability. To the best of our knowledge, Romano et al. (11) conducted the only study to date investigating the sustainability implications of wheat cultivation, which provides straw as a significant feed ingredient, particularly for dry buffaloes. However, poor nutritive value of WS limits its utilization for enhancing animal production. Strategic supplementation of feed resources could improve the fermentation and utilization of WS, thereby improving animal productivity (12). Many researchers demonstrated that supplementation of various local agricultural and agro-industrial by-products, oil cakes, or tree leaves could enhance the rumen function and utilization of fibrous feeds.

Sugar beet (*Beta vulgaris* L.) is a cash crop produced all over the world for sugar production by the industries. After extraction of sugar, the remaining residue, called ‘sugar beet pulp’, is an agricultural by-product and a rich source of residual sugar and digestible fiber. Studies demonstrated that the partial replacement of corn grain with SBP in the diet of cows or sheep enhanced rumen fermentation, milk production and feed efficiency (13–15). Experiments with Korean cattle steers and Merino lambs on partial replacement of either corn or barley grain with SBP described improved ruminal fermentation without affecting animal performance (16, 17). As WS serves as the major roughage source for resource-poor farmers in Asian countries, enhancing its utilization could strengthen the productivity of ruminants of this region. We hypothesized that the supplementation of SBP to a WS-based diet would modulate rumen fermentation by supplying highly fermentable carbohydrates to rumen microbes, thereby improving the utilization of WS. However, whether and how supplementation of SBP affects rumen fermentation kinetics, digestibility, fatty acid production and ruminal enzyme activities during fermentation of WS has not been reported. The current study, therefore, was designed to examine the influence of SBP in modulating rumen fermentation kinetics, volatile fatty acid production, enzyme activities, and methanogenesis of WS in the rumen liquor of water buffalo (*Bubalus bubalis*).

## 2 Materials and methods

### 2.1 Substrates

SBP was procured from M/s Rana Sugars Limited, Chandigarh, India (18) and ground to a particle size of 1 mm after drying in a hot-air oven. WS was procured from the institute’s field, dried and ground for use in an *in vitro* fermentation study. Representative samples for both feeds were taken for chemical analysis.

### 2.2 Rumen fluid collection

Fluid from the solid and liquid portions of the rumen was obtained from three Murrah buffalo steers, in accordance with Kamra and Agarwal (19), which were fitted with permanent cannulas, maintained at the institute’s Animal Nutrition Shed, and kept on a straw-based diet with a limited quantity of concentrate mixture and fodder oats in the morning (09:30 h) for maintenance requirements (10). All the methods were performed in accordance with the approval (IAEC-CIRB/2022 dated 4 July 2022) of the Institute’s Animal Ethics Committee (IAEC) following the relevant guidelines and regulations of the Committee for Control and Supervision of Experiments on Animals (CCSEA), Ministry of Fisheries, Animal Husbandry and Dairying, Department of Animal Husbandry and Dairying, Government of India (www.ccsea.gov.in) for conducting experiments using rumen fluid from buffalo steers. The collected rumen liquor was filtered over four sheets of cheesecloth. Approximately equal quantities of rumen fluid from each animal were collected and mixed in a thermos flask prewarmed and flushed with CO₂_._ The collected rumen fluid in the thermos flask was transported immediately to the Rumen Microbiome laboratory, where further studies were conducted under anaerobic conditions.

### 2.3 *In vitro* fermentation and gas production

The investigation of *in vitro* fermentation kinetics was carried out using the ANKOM RF radio-frequency-based gas production system (ANKOM Technology, Macedon, NY 14502, USA; www.ankom.com). Mineral solution mixed with buffer was prepared as per Menke and Steingass (20). Strained rumen liquor (SRL) was mixed (1:2) with buffer solution under anaerobic conditions by continuous influx of CO₂ to make an incubation medium, from which 60 mL was dispensed in 250 mL calibrated ANKOM bottles containing 500 mg test substrate. Four treatments were examined, in which SBP was incorporated at various amounts with WS to formulate four different diets in the following mixtures: T_1_: WS: SBP, 100:0; T_2_: WS: SBP, 0:100; T_3_: WS: SBP, 90:10; T_4_: WS: SBP, 80:20. The SBP supplementation dose levels (10–20%) to WS were hypothesized based on a practical feeding approach, preliminary studies in the authors’ laboratory, local availability, and supporting studies in other species (16, 21). The bottles were securely fitted with ANKOM RF components and pressure transducers for wireless data transmission, with the base controller attached to a computer, and the bottles containing samples and buffered rumen fluid were incubated in an incubator (Narang Scientific Works Pvt. Ltd., New Delhi, India, www.nsw-india.com) at 39 °C for 72 h with shaking at 85 oscillations/min. Only buffered rumen fluid was kept in three replicate bottles and incubated as a blank in each experiment. Three independent incubations were performed with five replicates at each time point for each treatment.

### 2.4 Estimation of total gas production and methane formation

Total gas production (TGP) was calculated using real-time measurements of temperature and gas pressure recorded by means of the ANKOM RF Gas Production System.

$$TGP\;\left(mol\right)=\left(p^{*}h\right)/\left(t^{*}a\right)$$

where.

*p* = pressure in the bottle (kPa);

h = volume of the headspace (mL);

t = temperature in the bottle (K);

a = gas constant (8.3144).

Gas produced in blank bottles over time was subtracted when calculating the total net production of gas on substrate fermentation (22). Methane concentration in headspace gas was determined using a gas chromatograph (NUCON-5700, Nucon Engineers, New Delhi, India) from all the bottles after termination of incubation at 24 and 72 h of fermentation. The Porapak-Q stainless-steel column and a flame ionization detector (FID) were used in the analysis. The gas chromatograph temperatures were maintained at 60 °C, 140 °C, and 150 °C for the column oven, injector, and detector, respectively, with a column pressure of 10 psi using hydrogen as the carrier gas. A sample of 200 μL of gas from the headspace of each bottle was taken manually using a Hamilton syringe and injected into the gas chromatograph calibrated with standard methane and carbon dioxide (23). A correction of net methane production from each substrate during every run was made by considering methane production in blank bottles. Each sample was studied for fermentation kinetics by conducting three separate runs. The total methane production from each substrate at every time point was calculated by multiplying the headspace methane concentration by the total gas production.

### 2.5 Estimation of volatile fatty acids (VFAs) in fermented fluid

One milliliter of the supernatant of the fermented fluid from each bottle at 24 and 72 h of incubation was transferred into microcentrifuge tubes containing 0.20 mL of 25% metaphosphoric acid. The mixture was kept at room temperature for 2 h and centrifuged at 5,000 × g for 10 min. The supernatant was collected in a microcentrifuge tube and kept at −20 °C for VFA determination (24). Volatile fatty acids were quantified using a gas chromatograph (NUCON-5700 Gas Chromatograph, Nucon Engineers, New Delhi, India; www.nuconengineers.com) fitted with a Chromosorb 101 glass column and a flame ionization detector (FID). For analysis, 1 μL of sample was injected manually into the gas chromatograph using a Hamilton syringe. The gas chromatograph was operated at pressures of 20 psi for hydrogen and 10 psi for moisture-free air, respectively. The oven temperature was programmed from 170 °C to 240 °C at 3 °C/min and held for 7 min. The temperatures of the injector and FID detector were fixed at 240 °C and 250 °C, respectively (25). The net VFA production was calculated by subtracting the VFA concentrations in blanks at specific time points.

### 2.6 Feed dry matter digestibility

At the termination of incubation (24 and 72 h), the contents of each bottle were quantitatively transferred to 500 mL spoutless beakers by repeated washings with neutral detergent solution (26). The beakers were refluxed for 1 h, and the residues were recovered by vacuum filtration through pre-weighed Borosil No. 1 porosity glass-fritted crucibles. The residues were subsequently washed with hot water, and the crucibles containing the fermentation residues were oven-dried at 105 °C for 24 h to constant weight for determination of dry matter.

### 2.7 Enzyme assay

After 24 h of incubation, the contents of each bottle were transferred to a conical flask containing carbon tetrachloride and lysozyme solutions for extraction of enzymes. The contents were processed as described earlier (27, 28) and the supernatant was used for the assessment of fibrolytic enzyme activities. The carboxymethyl cellulase (CMCase) and xylanase activities were quantified by assessing released reducing sugars in the reaction mixture (28) using standards of glucose (Himedia Laboratories Limited, India) and xylose (Sigma Aldrich Limited, New Delhi, India). The samples were analyzed for protein content (29) and the enzyme activities (mIU) were expressed as nanomoles of reducing sugars released per unit time.

### 2.8 Chemical analysis

The samples of feeds, namely WS and SBP, were analyzed in triplicate following the methods of AOAC (30) to determine dry matter (DM) by the oven-drying method, organic matter (OM) by muffle-furnace incineration, and crude protein (CP) by the Kjeldahl method. Approximately 1–2 g of the feed sample was digested with 30 mL of concentrated sulfuric acid in the presence of a digestion mixture (5.0g; CuSO4: K_2_SO_4_, 1:9) using a Kjeldahl flask. After digestion, the clear digest was allowed to cool and quantitatively transferred to a 250 mL volumetric flask with repeated rinsing using distilled water. An aliquot of 10 mL was then distilled in a Kjeldahl distillation unit, and the liberated ammonia was trapped in 3% boric acid solution containing Tashiro’s indicator. The collected distillate was titrated against 0.01 N H₂SO₄ and the crude protein content was calculated using the following equation:

$$CP=\left(tv^{*}b^{*}0.014^{*}6.25\right)/\left(c^{*}a\right)$$

where.

C*P* = crude protein (%).

tv = titer volume (mL) of 0.01 N H₂SO₄.

b = total volume (mL) made from the sample.

c = weight (g) of the sample taken for digestion on a DM basis.

a = aliquot taken (mL) for the distillation.

Neutral detergent fiber (NDF) and acid detergent fiber (ADF) were determined (26) without sodium sulfite and *α*-amylase. Volatile fatty acids were estimated (25) using gas chromatography.

### 2.9 Calculations for predicting *in vivo* digestibility of organic matter and metabolizable energy

Truly degradable dry matter (TDDM) was determined by subtracting the residue weight after treatment with NDS from the incubated dry matter (31). The net gas produced after feed fermentation at both 24 and 72 h, in combination with other chemical constituents of the feeds, was used to predict *in vivo* digestibility of organic matter and metabolizable energy (31).

ME=2.20+0.136gp+0.057cp

OMD=14.88+0.889gp+0.45cp+0.0651xa

where.

ME = metabolizable energy (MJ/kg DM).

OMD = organic matter digestibility (%).

c*p* = crude protein (%).

xa = ash (%).

g*p* = net gas production (mL/200 mg dry sample over time, i.e., after 24 and 72 h of incubation).

### 2.10 Statistical analyses

A nonlinear model was used to describe *in vitro* gas production kinetics and estimate the potential extent (*b*) and fractional rate (*c*) of fermentation from cumulative gas production data. The rate and extent of gas production were estimated by fitting cumulative gas production data to the nonlinear model of Orskov and McDonald (32):

$$Y_{t}=b\;\left(1-e^{-ct}\right)$$

where.

*Y_t_* = cumulative gas production (mL/g DM) at incubation time t (h),

*b =* asymptotic or potential gas production (mL/g DM),

*c* = fractional rate constant of gas production (/h),

*t* = incubation time (h),

*e* = base of the natural logarithm.

The values of *b* and *c* were assessed by an iterative least-squares procedure using a nonlinear regression method of IBM SPSS Statistics (33). Five replicate bottles within each incubation run were averaged and the three incubation runs (n = 3) for each treatment were the statistical units. Repeated-measures ANOVA was performed for the gas production data obtained at various time points with treatment as the between-subject factor and time as the within-subject factor, and treatment × time as the interaction term. For other parameters, the data obtained at 24 h and 72 h were derived from separate incubation bottles and were analyzed independently through one-way analysis of variance (ANOVA) using the general linear model procedure (16) in a completely randomized design with treatment as the fixed effect for each incubation time to compare treatment means. The normality of data distribution was verified using the UNIVARIATE procedure of IBM SPSS Statistics (Version 20.0). Differences among means were assessed using Tukey’s honestly significant difference (HSD) test (34), and statistical significance was declared at *p* ≤ 0.05. Linear regression analysis was performed, where methane production was used as the dependent variable, while truly degradable dry matter, individual volatile fatty acids (VFAs) and their ratios were used as independent variables. Multicollinearity was assessed using the variance inflation factor (VIF). Model assumptions of normality and homoscedasticity were verified through residual diagnostics. Multivariable regression or multivariate approaches could provide a more integrated assessment of the relationships among fermentation variables. However, considering the limited sample size and the potential for multicollinearity among the fermentation parameters, such models may yield unstable parameter estimates and complicate biological interpretation. Therefore, simple regression analyses were performed for the exploratory purposes of the present study.

## 3 Results

SBP contained 9.74% crude protein, 60.65% neutral detergent fiber, and 31.02% acid detergent fiber (Table 1), indicating its potential as a source of nutrients and fiber for animals.

**Table 1:** Proximate composition and fiber fractions (% DM) of sugar beet pulp and wheat straw.

Description	OM	Ash	CP	EE	NDF	ADF
Wheat Straw	92.55	7.45^a^	3.80^a^	1.30^b^	84.20^b^	62.25^b^
Sugar Beet Pulp	92.02	7.98^b^	9.74^b^	0.82^a^	60.65^a^	31.02^a^
SEM	0.12	0.13	1.32	0.11	5.02	6.98
P-value	0.608	0.014	<0.001	<0.001	<0.001	<0.001

OM, organic matter; CP, crude protein; EE, ether extract; NDF, neutral detergent fiber; ADF, acid detergent fiber; SEM, standard error of the mean. Values with different superscripts (a, b) in the same column differ significantly (*p* < 0.05).

At the very early stage (2 h) of the fermentation process, SBP20 showed the highest gas production; however, at 4 h of fermentation, it remained comparable (*p* > 0.05) with SBP alone. The gas production at 72 h of fermentation was highest (*p* < 0.01) with SBP alone, whereas it was lowest (*p* < 0.01) with WS alone. Compared to WS alone (Control), supplementation of SBP either at 10% (SBP10) or 20% (SBP20) to WS enhanced (*p* < 0.01) total gas production at all time points; however, gas production remained intermediate between that of SBP and WS. SBP exhibited the highest (*p* < 0.01) potential gas production (*b*) and net gas production (Table 2) at 72 h, whereas WS was lowest. Supplementation with SBP enhanced the potential gas production (*b*, mL/g DM) of composite feeds of WS and SBP, although the increase was greater (*p* < 0.01) in SBP20 than SBP10. The rate (per h) of gas production (*c*) was highest (*p* < 0.01) in the SBP20 group, followed by SBP, SBP10, and WS; however, SBP10 remained comparable (*p* > 0.05) with SBP.

**Table 2:** Effects of sugar beet pulp supplementation on *in vitro* gas production kinetics of wheat straw.

Description	Gas Produced (mL/g DM)							Gas production constant	
	2 h	4 h	8 h	12 h	24 h	48 h	72 h	b (mL/g DM)	c (rate/h)
WS	19.96^a^	26.00^a^	36.54^a^	41.98^a^	64.68^a^	82.84^a^	77.53^a^	81.82^a^	0.07^a^
SBP	37.15^c^	63.92^c^	108.70^d^	158.09^d^	216.38^d^	221.27^d^	198.21^d^	216.92^d^	0.10^b^
SBP10	24.81^b^	36.46^b^	52.63^b^	63.45^b^	88.06^b^	103.95^b^	99.42^b^	103.13^b^	0.091^b^
SBP20	53.82^d^	66.19^c^	85.85^c^	94.15^c^	120.68^c^	125.22^c^	120.18^c^	118.96^c^	0.191^c^
SEM	3.49	5.19	8.78	12.10	14.73	14.24	12.19	19.57	0.017
Source of variation	*P*-value								
Treatment	<0.001								
Time	<0.001								
Treatment × time	<0.001								

WS, wheat straw; DM, dry matter; SEM, standard error of the mean; b, potential gas production; c, fractional rate of gas production; SBP, sugar beet pulp; SBP10, 90% WS and 10% SBP; SBP20, 80% WS and 20% SBP. Values with different superscripts (a, b, c, d) in the same column differ significantly (*P* < 0.001).

There were differences (*p* < 0.05) in the estimated true degradability of dry matter (TDDM) and calculated digestibility of organic matter (OMD) and metabolizable energy (ME) content of WS, SBP, and their composite feeds at both 24 h and 72 h of fermentation (Table 3). SBP exhibited higher (*p* < 0.01) TDDM, OMD, and ME than the other feeds (Table 3). The TDDM content of WS was estimated to be the lowest (*p* < 0.05) among all the feeds examined, resulting in lower OMD and ME contents. Supplementation of WS with SBP enhanced (*p* < 0.05) the TDDM, OMD, and ME contents of composite feeds. Although TDDM varied (*p* < 0.05) between SBP10 and SBP20, the OMD and ME values remained comparable (*p* > 0.05).

**Table 3:** *In vitro* true digestibility (TDDM) and methane production of wheat straw with graded levels of sugar beet pulp supplementation over time.

Description	TDDM (%)		Methane (mL/g DM)		Methane (mL/g DDM)		OMD (%)		ME (MJ/kg DM)	
	24 h	72 h	24 h	72 h	24 h	72 h	24 h	72 h	24 h	72 h
WS	27.54^a^	56.53^a^	2.08^a^	3.55^a^	2.23^a^	6.27^a^	28.18^a^	30.39^a^	4.12^a^	4.45^a^
SBP	59.28^d^	92.43^d^	8.56^c^	9.02^b^	3.56^b^	9.79^b^	58.26^d^	55.02^c^	8.64^c^	8.15^c^
SBP10	48.32^b^	60.21^b^	1.98^a^	3.15^a^	2.12^a^	5.29^a^	32.52^b^	34.48^b^	4.77^ab^	5.07^ab^
SBP20	52.75^c^	63.12^c^	2.22^b^	3.42^a^	2.32^a^	5.47^a^	38.49^c^	38.40^b^	5.68^b^	5.66^b^
SEM	2.12	3.66	0.64	0.72	0.25	0.56	2.16	3.05	0.23	0.38
P-value	0.03	<0.01	0.02	<0.01	0.02	<0.01	<0.01	<0.01	<0.01	<0.01

WS, wheat straw; TDDM, truly degradable dry matter; DDM, digestible dry matter; OMD, organic matter digestibility; ME, metabolizable energy; DM, dry matter; SEM, standard error of the mean; SBP, sugar beet pulp; SBP10, 90% WS and 10% SBP; SBP20, 80% WS and 20% SBP. Values with different superscripts (a, b, c, d) in the same column differ significantly (*P* < 0.05).

As fermentable carbohydrates are less abundant in WS, its TDDM as well as methane production were lower (*p* < 0.01) than those of SBP at 72 h of fermentation (Table 3). Although degradability of the composite feeds was increased (*p* < 0.01) due to SBP supplementation, irrespective of the supplementation level, the methane production (mL/g DM incubated or mL/g digestible DM) remained comparable (*p* > 0.05) with WS after 72 h of fermentation.

The production of major volatile fatty acids including acetate, propionate, and butyrate was highest with SBP alone followed by SBP20, SBP10, and WS (Table 4) at both time points of 24 h (*p* < 0.05) and 72 h (*p* < 0.01). Supplementation of SBP at both levels (SBP10 and SBP20) increased (*p* < 0.01) fatty acid production compared with WS alone; however, production was similar (*p* > 0.05) between SBP10 and SBP20. The A: P ratio was lowest (*p* < 0.01) in SBP after 72 h of fermentation compared with the other treatments.

**Table 4:** Volatile fatty acid production (mM/100 mL) during incubation of wheat straw and sugar beet pulp with buffered rumen fluid.

Description	Acetate		Propionate		Butyrate		A: P ratio	
	24 h	72 h	24 h	72 h	24 h	72 h	24 h	72 h
WS	2.60^a^	4.91^a^	0.98^a^	1.25^a^	0.32^a^	0.58^a^	2.65^a^	3.93^b^
SBP	6.01^c^	9.95^c^	1.74^c^	3.23^c^	0.89^c^	1.33^c^	3.46^b^	3.08^a^
SBP10	5.12^b^	7.19^b^	1.25^ab^	2.03^b^	0.65^b^	0.94^b^	4.10^c^	3.53^b^
SBP20	5.38^b^	7.53^b^	1.36^b^	2.18^b^	0.73^b^	1.00^b^	3.96^c^	3.45^b^
SEM	0.22	0.31	0.09	0.12	0.11	0.05	0.06	0.05
P-value	0.02	<0.01	0.04	<0.01	0.03	<0.01	<0.01	<0.01

A:P, acetate-to-propionate ratio; WS, wheat straw; SEM, standard error of the mean; SBP, sugar beet pulp; SBP10, 90% WS and 10% SBP; SBP20, 80% WS and 20% SBP. Values with different superscripts (a, b, c) in the same column differ significantly (*P* < 0.05).

The CMCase and xylanase activities were higher (*p* < 0.01) in SBP than in the WS group. Although CMCase (*p* < 0.01) and xylanase (*p* < 0.05) activities increased with the inclusion of SBP at both levels compared with WS, SBP10 and SBP20 remained comparable (*p* > 0.05) (Table 5).

**Table 5:** Fibrolytic enzyme activities (mIU/mg protein) during incubation of wheat straw with graded levels of sugar beet pulp.

Description	CMCase	Xylanase
Wheat straw	219.45^a^	620.26^a^
Sugar Beet Pulp	413.89^b^	697.49^c^
SBP10	308.26^c^	660.42^b^
SBP20	348.38^c^	679.14^bc^
SEM	22.56	11.29
*P*-value	0.004	0.03

CMCase, carboxymethyl cellulase; WS, wheat straw; SEM, standard error of the mean; SBP, sugar beet pulp; SBP10, 90% WS and 10% SBP; SBP20, 80% WS and 20% SBP. Values with different superscripts (a, b, c) in the same column differ significantly (*P* < 0.05).

The linear regression analysis (Table 6) revealed a strong positive association between methane production (mL/g DM) and TDDM (R^2^ = 0.949, *p* < 0.001), whereas methane production was negatively correlated with acetate-to-propionate ratio (R^2^ = 0.602, *p* < 0.01). Methane production also exhibited significant positive associations with acetate and butyrate concentrations (*p* < 0.01). Although propionate is generally considered a competitive hydrogen sink, a positive relationship between methane production and propionate concentration was observed (R^2^ = 0.680, *p* < 0.01), likely reflecting enhanced overall fermentation activity, particularly in response to sugar beet pulp supplementation.

**Table 6:** Regression analysis of methane production (mL/g DM) as influenced by fermentation parameters.

Predictor variable	R^2^	B	β	*F*-value	P-value
TDDM (%)	0.949	0.168	0.974	185.58	<0.001
A: P ratio	0.602	–6.357	–0.776	15.144	0.003
Acetate (mM/dL)	0.602	1.053	0.776	15.126	0.003
Propionate (mM/dL)	0.680	2.886	0.825	21.298	0.001
Butyrate (mM/dL)	0.567	6.864	0.753	13.101	0.005

TDDM, truly degradable dry matter; DM, dry matter; A:P ratio, acetate-to-propionate ratio; R^2^, coefficient of determination; B, unstandardized coefficient; β, standardized coefficient.

## 4 Discussion

**Figure 1:**
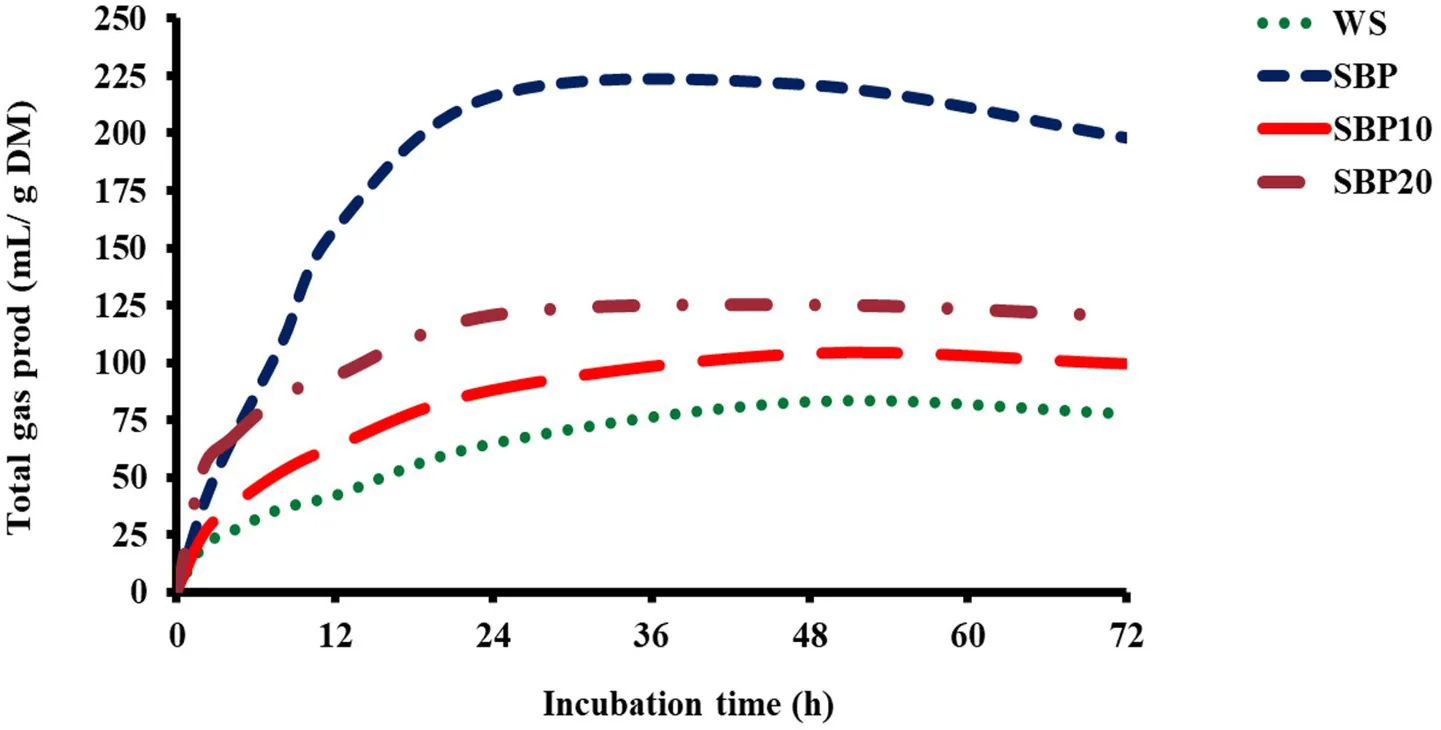
Cumulative *in vitro* gas production profiles of wheat straw, sugar beet pulp, and their combinations during incubation in buffered rumen fluid of buffaloes. WS, wheat straw; SBP, sugar beet pulp; SBP10, 10% SBP and 90% WS; SBP20, 20% SBP and 80% WS.

SBP is a suitable source of energy with highly digestible fiber content (35), whereas WS is rich in lignocellulosic biomass (2, 36). The fermentation of WS is low due to lignocellulosic bonds, which rumen bacteria, protozoa, and fungi have limited access to (37, 38). Although the chemical composition of agricultural by-products varies with the cultivar., maturity, and leaf-to-stem ratio, the present study demonstrated the nutritional value of WS and SBP (Table 1) in agreement with earlier studies (13, 39–42). Gas production results from microbial fermentation of carbohydrates to volatile fatty acids (22, 31). As the proximate compositions and fiber fractions of the WS, SBP, and composite feeds varied, the rate of gas production at various time points (Table 2) and net total gas production at 72 h differed (Figure 1). Net gas production increased (*p* < 0.001) in SBP and was lowest in WS, whereas it was intermediate in WS supplemented with SBP (SBP10 and SBP20) (Figure 1). SBP contained more hemicelluloses (Table 1) and other fermentable carbohydrates as well as less ADF, which supplied the substrates for the microbes present in the rumen, demonstrating increased gas production (Figure 1). As in the present study, earlier researchers (43, 44) also reported an inverse relationship between gas production and fiber content in the feeds. In an *in vitro* study on graded levels of supplementation of fodder beet root to perennial ryegrass, Fleming et al. (45) demonstrated a linear increase in cumulative gas production owing to modulation of fermentation kinetics through the supply of readily fermentable carbohydrates to the rumen microbes.

The higher values for TDDM, OMD, and ME in SBP (Table 3) could be owing to an enhanced rate of fermentation and the presence of soluble carbohydrates. SBP is a by-product of the sugar-processing industry, which contains pectin as a major part of fiber (41, 46) and is reported to be degraded rapidly in the rumen (47, 48). As WS is rich in lignocellulosic materials, the digestibility values are lower (37, 49). Strategic supplementation of WS with grains, tree leaves (*Moringa oleifera, Morus alba, Vigna mungo*), or molasses (21, 50) was reported to increase the digestibility and nutritive value of WS due to the availability of readily fermentable carbohydrates, which stimulate the activity of rumen microbes (51, 52). The present study also demonstrated enhanced digestibility (Table 3) and ME content of composite feeds, in which WS was supplemented with graded levels of SBP (SBP10 and SBP20). Consistent with our study, Abo-Donia et al. (53) reported that feeding ensiled rice straw with sugar beet enhanced composite-feed digestibility and milk production.

**Figure 2:**
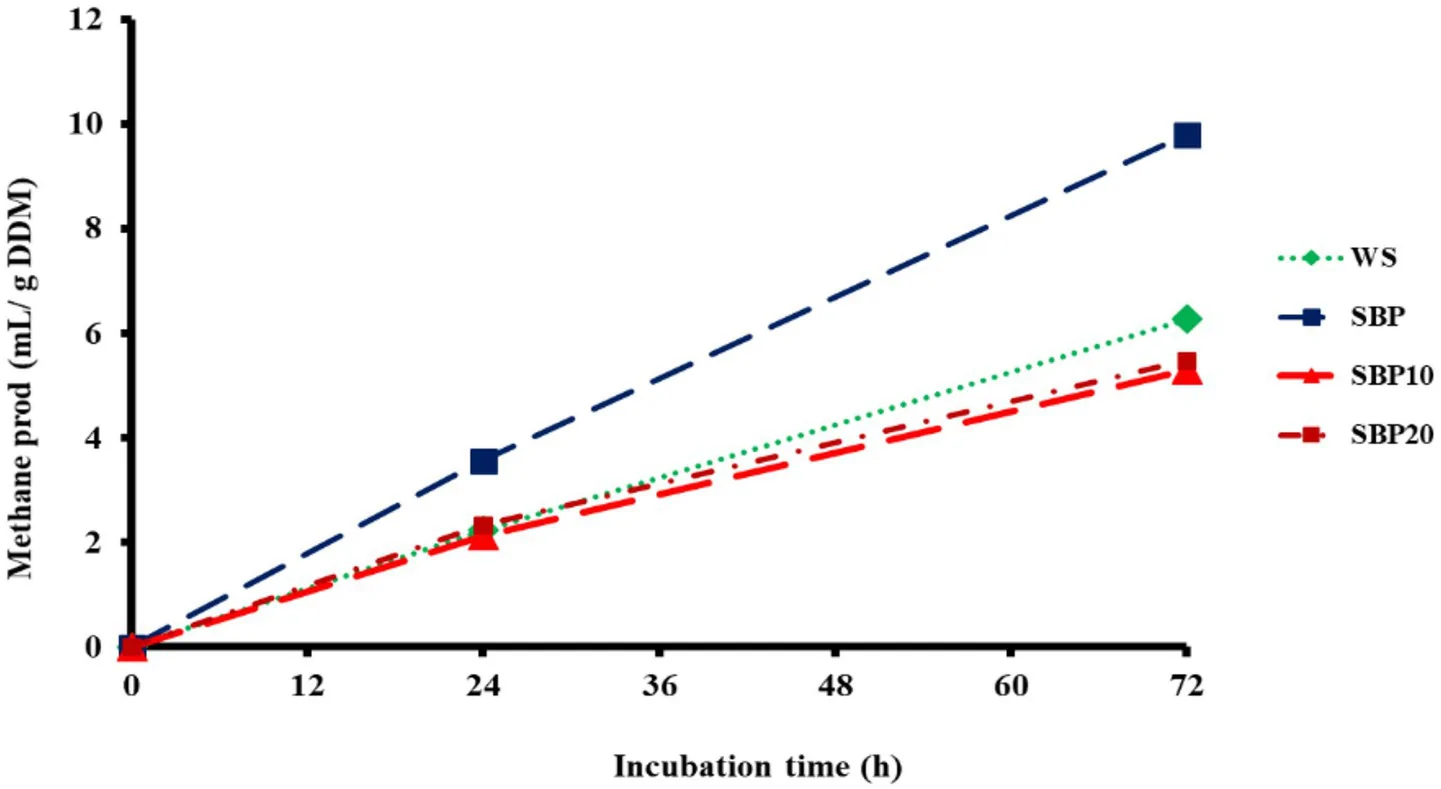
Comparative methane production during *in vitro* fermentation of wheat straw, sugar beet pulp, and their combinations in buffered rumen fluid from buffaloes. WS, wheat straw; SBP, sugar beet pulp; SBP10, 10% SBP and 90% WS; SBP20, 20% SBP and 80% WS.

The anaerobic fermentation of carbohydrates in the gut of herbivores by a mixed microbial population (41) produces volatile fatty acids, which are energy sources for animals, with methane as a by-product. As methane production depends on the digestibility and rate of fermentation of feeds (54, 55), methane production remained lower in WS and higher in SBP. However, strategic supplementation of WS with SBP demonstrated a tendency (*p* = 0.085) toward comparable methane production (Figure 2); the enhanced digestibility (Table 3) could be due to better utilization of feeds for volatile fatty acid production (56, 57).

Higher (*p* < 0.01) production of volatile fatty acids (acetate, propionate, and butyrate) with SBP could be due to the nature of the cell wall and enhanced digestibility of SBP fiber, whereas lower VFA production from WS resulted from complex lignocellulosic structure of the cell wall, which has poor digestibility (Table 3). Supplementation of WS with SBP enhanced digestibility of the composite feed, thereby increasing VFA production (Table 4). In a study with Holstein dairy cows, Mohsen et al. (13) demonstrated enhanced ruminal fatty acid production after dietary replacement of yellow corn with graded levels of dried SBP. As in the present study, increased production of volatile fatty acids was also reported in lambs with supplementation of SBP (58).

Ruminal enzyme activities depend on the composition of diets fed to the animals (59). The enhanced activity of CMCase and xylanase on *in vitro* ruminal incubation of SBP and a composite feed of WS supplemented with SBP could be due to increased availability of fermentable carbohydrates (Table 1) resulting in greater microbial colonization, also confirmed by the enhanced digestibility and gas production (60). In cattle fed a wheat straw-based diet, supplementation with concentrate feeds increased fiber-degrading enzyme activity. Santra and Pathak (61) reported that concentrations of polysaccharide-degrading enzymes depend on substrate availability. The statistically validated regression analyses demonstrated that digestibility-driven fermentation intensity was the primary determinant of methane production, with TDDM as the strongest single predictor of methane. Increased digestibility leads to greater microbial activity with higher hydrogen availability and methane production. The inverse relationship of the A:P ratio indicated the diversion of hydrogen toward propionate production, reducing hydrogen availability for methanogens. The overall increase in the intensity of fermentation owing to supplementation of SBP with WS was responsible for higher acetate and propionate production. Although propionate is usually considered a hydrogen sink, under *in vitro* conditions, the positive association with methane production could be due to greater substrate degradation, modulating rumen fermentation, thereby increasing total gas and methane production. In support of our study, an experiment on cannulated sheep with different feeding modules by Robinson et al. (62) reported higher acetate and propionate concentrations with enhanced daily methane production, and none of the VFA parameters offered a useful tool to predict methane production. Our study further indicated that VFA concentration acts as a secondary modulator, whereas feed digestibility is the primary determinant of methane production. Other researchers (63–66), while experimenting on lactating cows with various feeding modules, reported no or a weak relationship of methane production with ruminal volatile fatty acids. Consistent with our study, Negussie et al. (67) concluded that the relationships between methane production and ruminal VFA were variable and not as straightforward as theory suggested.

## 5 Conclusion

Crop residues such as straws and stovers constitute an important component of livestock diets in smallholder farming systems across Asian countries. However, the low digestibility of these agricultural by-products limits nutrient utilization and consequently reduces animal performance. Supplementation of easily fermentable feeds could be a strategy for enhancing rumen function, thereby improving the utilization of WS. SBP, a widely available by-product of the sugar industry, could be an advisable choice to enhance the utilization of WS. Although SBP, as an agro-industrial by-product, is available at low cost in comparison to other energy sources, the implementation of its strategic supplementation for enhancing the utilization of WS in a practical feeding system would largely depend on the local cost and availability. The present findings demonstrated enhanced digestibility, ruminal fatty acid production, and enzyme activities from composite diets when WS was supplemented with 10–20% SBP, indicating better utilization of WS by animals. However, *in vivo* studies in growing and lactating buffaloes are warranted to examine production performance before actual field implementation in developing countries.

## Data Availability

The original contributions presented in the study are included in the article/supplementary material, further inquiries can be directed to the corresponding author/s.
